# Supplementary figures and images for: Planting time shapes fall armyworm infestation dynamics and associated yield loss of maize in Bangladesh
Source: PLoS One. 2026 Apr 15;21(4):e0347125. doi: 10.1371/journal.pone.0347125 (PMC13082657; doi:10.1371/journal.pone.0347125)

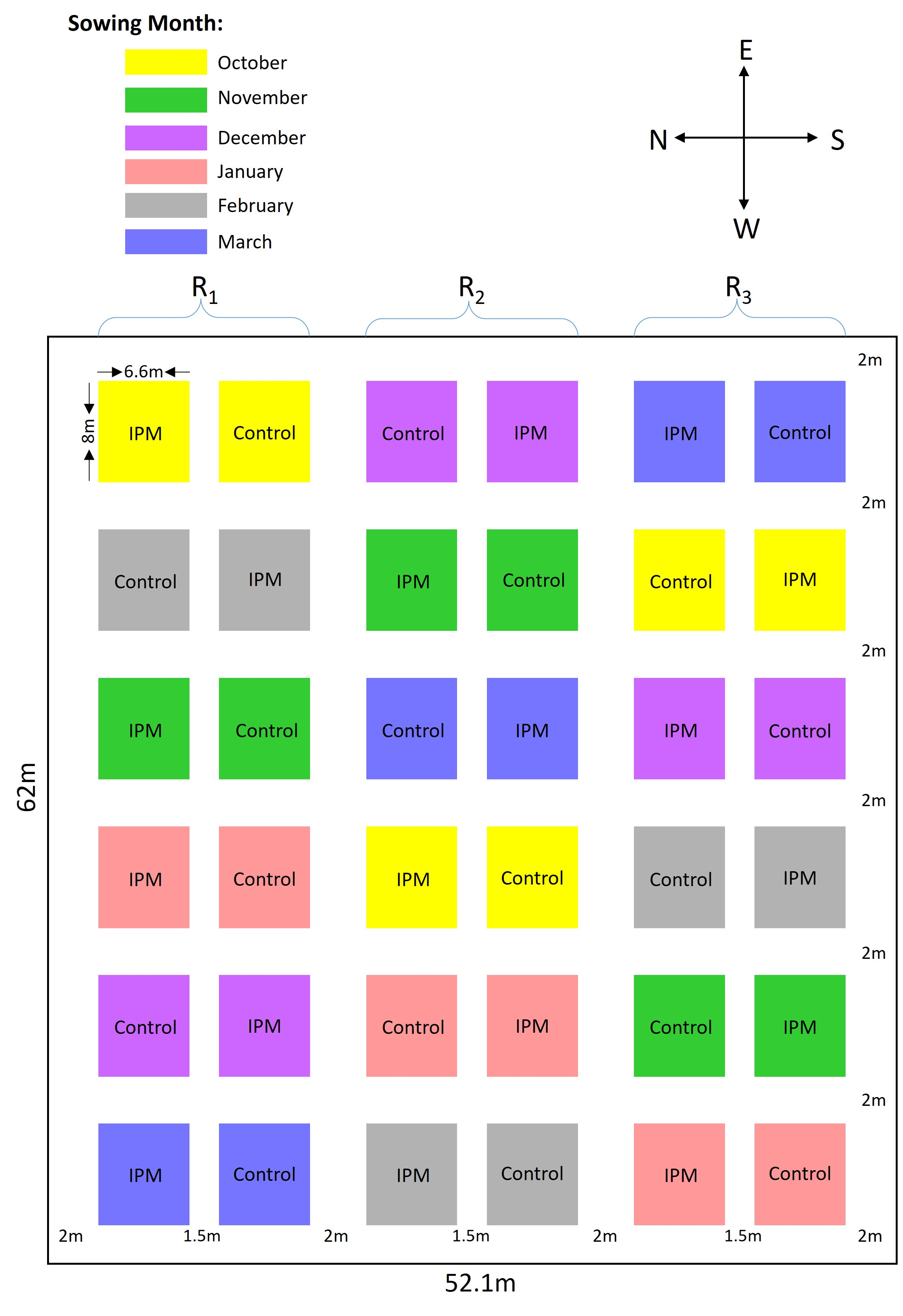

Supplement: S1 Fig — (TIF) [file pone.0347125.s001.tif]

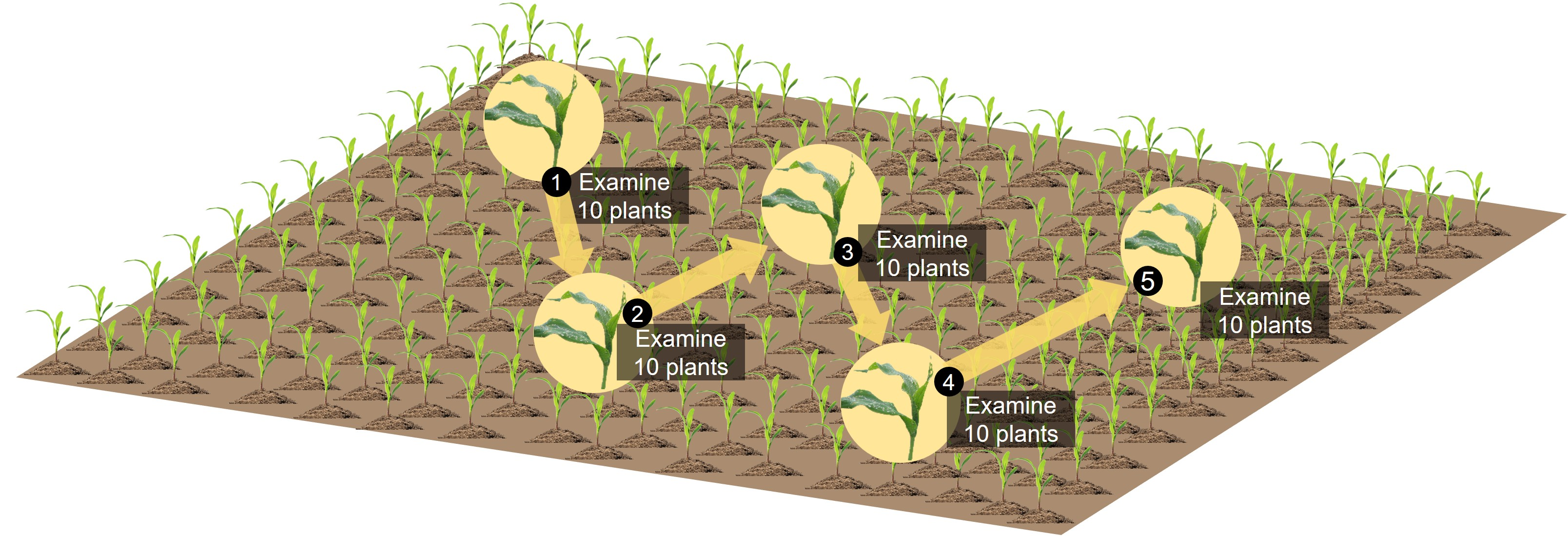

Supplement: S2 Fig — (TIF) [file pone.0347125.s002.tif]

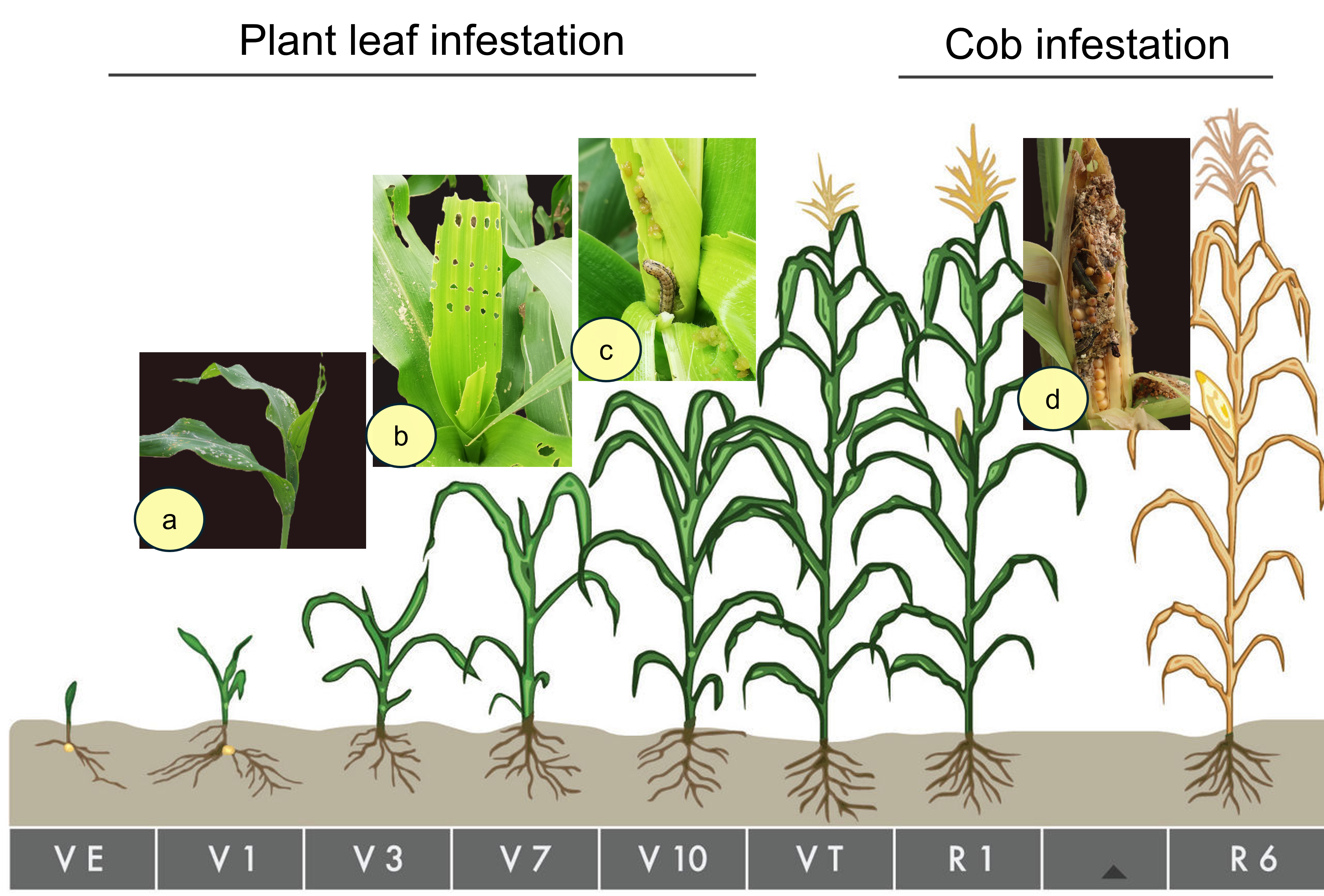

Supplement: S1 File — Feeding pattern: (a) Small Fresh Windowing (SFW) by 1–2 instar larvae (b) Ragged linear holes by 3–4 instar larvae (c) Infested Whorl (IW) caused by mostly 5–6 instar larvae, and (d) Enormous cob damage by 1–6 instar larvae. (TIF) [file pone.0347125.s006.tif]
