# Supplementary material for: Planting time shapes fall armyworm infestation dynamics and associated yield loss of maize in Bangladesh
Source: PLoS One. 2026 Apr 15;21(4):e0347125. doi: 10.1371/journal.pone.0347125 (PMC13082657; doi:10.1371/journal.pone.0347125)
Supplement: S1 Table — (DOCX) [file pone.0347125.s003.docx]

**S1 Table.** Mean (± SE) percent leaf infestation in maize by *Spodoptera frugiperda* as influenced by Month × Treatment interaction, with means grouped using Tukey’s HSD test

| **Sowing month** | **Treatment** | **Maize growth stages** | | | | |
| --- | --- | --- | --- | --- | --- | --- |
|  |  | **V4** | **V6** | **V8** | **V10** | **V12** |
| October | Control | 52.44 ± 5.20 a | 68.00 ± 3.55 a | 50.22 ± 4.36 b | 42.67 ± 3.75 c | 43.11 ± 4.4 b |
|  | IPM | 14.22 ± 1.64 b-d | 8.00 ± 1.79 de | 4.89 ± 1.26 d-f | 1.11 ± 0.47 f | 0.89 ± 0.43 e |
| November | Control | 20.89 ± 2.37 b | 13.11 ± 1.65 d | 18.00 ± 2.10 c | 15.56 ± 2.24 de | 12.22 ± 1.71 cd |
|  | IPM | 4.4 ± 0.98 de | 1.78 ± 0.73 e | 4.89 ± 1.33 d-f | 1.56 ± 0.55 f | 1.33 ± 0.51 de |
| December | Control | 10.44 ± 1.56 b-e | 11.33 ± 1.90 de | 12.89 ± 2.22 c-f | 9.11 ± 1.65 ef | 18.44 ± 3.16 c |
|  | IPM | 1.78 ± 0.66 e | 1.33 ± 0.60 e | 3.33 ± 1.01 ef | 5.33 ± 1.17 ef | 6.22 ± 1.57 de |
| January | Control | 8.89 ± 1.69 c-e | 7.11 ± 1.37 de | 15.78 ± 3.04 cd | 20.67 ± 2.19 d | 11.11 ± 1.28 c-e |
|  | IPM | 1.78 ± 0.58 e | 1.11 ± 0.47 e | 2.67 ± 0.97 f | 2.44 ± 0.85 f | 6.89 ± 1.09 de |
| February | Control | 16.67 ± 1.93 bc | 50.22 ± 2.72 b | 63.11 ± 3.61 a | 63.33 ± 4.24 b | 86.00 ± 1.63 a |
|  | IPM | 10.44 ± 1.56 b-e | 26.89 ± 3.80 c | 16.67 ± 3.23 cd | 8.44 ± 1.91 ef | 5.78 ± 1.54 de |
| March | Control | 47.11 ± 4.20 a | 61.56 ± 3.24 a | 74.67 ± 4.14 a | 79.11 ± 4.49 a | 78.22 ± 4.55 a |
|  | IPM | 20.67 ± 2.89 b | 16.22 ± 1.81 d | 15.11 ± 2.15 c-e | 11.56 ± 2.20 d-f | 6.89 ± 1.88 de |
| F_5,524_ | | 14.57*** | 48.65*** | 33.61*** | 51.80*** | 98.50*** |

DF (df1, df2) indicate degrees of freedom as the numerator and the denominator; *** indicates *P* < 0.0001; Values in columns not separated by sources of variation and comprising the same letter are not significantly differed according to Tukey’s HSD at α = 0.05.
