# Supplementary material for: Planting time shapes fall armyworm infestation dynamics and associated yield loss of maize in Bangladesh
Source: PLoS One. 2026 Apr 15;21(4):e0347125. doi: 10.1371/journal.pone.0347125 (PMC13082657; doi:10.1371/journal.pone.0347125)
Supplement: S2 Table — (DOCX) [file pone.0347125.s004.docx]

**S2 Table.** ANOVA results (mean ± SE) of leaf infestation across planting months and treatments of different vegetative growth stages in maize under *Spodoptera frugiperda* infestation, with means grouped using Tukey’s HSD test

| **Variables** | **V4** | **V6** | **V8** | **V10** | | | **V12** |
| --- | --- | --- | --- | --- | --- | --- | --- |
| **Month** | | | | | | | |
| October | 33.33 ± 3.39 a | 38.00 ± 3.74 a | 27.56 ± 3.3 b | | 21.89 ± 2.9 c | 22.00 ± 3.14 b | |
| November | 12.67 ± 1.55 bc | 7.44 ± 1.08 b | 11.44 ± 1.42 c | | 8.56 ± 1.36 d | 6.78 ± 1.06 c | |
| December | 6.11 ± 0.96 cd | 6.33 ± 1.12 b | 8.11 ± 1.31 c | | 7.22 ± 1.02 d | 12.33 ± 1.87 c | |
| January | 5.33 ± 0.96 d | 4.11 ± 0.79 b | 9.22 ± 1.73 c | | 11.56 ± 1.51 d | 9.00 ± 0.87 c | |
| February | 13.56 ± 1.28 b | 38.56 ± 2.63 a | 39.89 ± 3.44 a | | 35.89 ± 3.72 b | 45.89 ± 4.4 a | |
| March | 33.89 ± 2.9 a | 38.89 ± 3.03 a | 44.89 ± 3.92 a | | 45.33 ± 4.36 a | 42.56 ± 4.5 a | |
| **Treatment** | | | | | |  | |
| IPM | 8.89 ± 0.77 b | 9.22 ± 1.85 b | 7.93 ± 0.82 b | | 5.07 ± 0.60 b | 4.67 ± 0.54 b | |
| Control | 26.07 ± 1.65 a | 35.22 ± 0.97 a | 39.11 ± 2.05 a | | 38.41 ± 2.06 a | 41.52 ± 2.25 a | |
| ***F-values*** | | | | | |  | |
| Month (F_5,524_) | 59.19*** | 128.68*** | 75.70*** | | 81.15*** | 107.12*** | |
| Treatment (F_1,524_) | 156.83*** | 409.71*** | 415.63*** | | 543.11*** | 737.12*** | |
| Month × Treatment (F_5,524)_ | 14.57*** | 48.65*** | 33.61*** | | 51.80*** | 98.50*** | |

DF (df1, df2) indicate degrees of freedom as the numerator and the denominator; *** indicates *P* < 0.0001, respectively; Values in columns not separated by sources of variation and comprising the same letter are not significantly differed according to Tukey’s HSD at α = 0.05.
