# Supplementary material for: Planting time shapes fall armyworm infestation dynamics and associated yield loss of maize in Bangladesh
Source: PLoS One. 2026 Apr 15;21(4):e0347125. doi: 10.1371/journal.pone.0347125 (PMC13082657; doi:10.1371/journal.pone.0347125)
Supplement: S3 Table — (DOCX) [file pone.0347125.s005.docx]

**S3 Table.** Mean (± SE) plant number, cob number, and yield of maize as influenced by the Month × Treatment interaction under *Spodoptera frugiperda* infestation, with means grouped using Tukey’s HSD test

| **Month** | **Treatment** | **Plant no./3.6m^2^** | **Cob no./3.6 m^2^** | **Yield (kg/ha)** |
| --- | --- | --- | --- | --- |
| October | IPM | 26.44 ± 0.53 a-c | 29.85 ± 1.49 | 14134.33 ± 637.67 a |
|  | Control | 26.63 ± 0.55 ab | 29.52 ± 1.44 | 12734.92 ± 577.92 bc |
| November | IPM | 25.40 ± 0.40 a-c | 30.33 ± 0.35 | 13244.40 ± 285.83 a-c |
|  | Control | 25.96 ± 0.35 a-c | 30.11 ± 0.53 | 12967.80 ± 247.55 a-c |
| December | IPM | 26.96 ± 0.29 ab | 27.07 ± 0.29 | 13889.03 ± 241.95 ab |
|  | Control | 26.85 ± 0.25 ab | 27.93 ± 0.25 | 13437.72 ± 247.55 a-c |
| January | IPM | 26.74 ± 0.37 ab | 27.89 ± 0.37 | 12821.74 ± 104.84 a-c |
|  | Control | 27.37 ± 0.33 a | 28.19 ± 0.36 | 12397.16 ± 88.51 c |
| February | IPM | 26.07 ± 0.42 a-c | 26.11 ± 0.43 | 10744.60 ± 161.04 d |
|  | Control | 25.07 ± 0.46 bc | 25.19 ± 0.47 | 8655.34 ± 135.05 e |
| March | IPM | 26.41 ± 0.37 a-c | 26.48 ± 0.37 | 8600.90 ± 243.70 e |
|  | Control | 24.56 ± 0.77 c | 24.52 ± 0.77 | 5917.27 ± 249.72 f |
| F_5,308_ | | 2.60* | 0.80^ns^ | 5.99^***^ |

DF (df1, df2) indicate degrees of freedom as the numerator and the denominator; * and *** indicate *P* < 0.05, and *P* < 0.0001, respectively; ns = non-significant; Values in columns not separated by sources of variation and comprising the same letter are not significantly differed according to Tukey’s HSD at α = 0.05.
