# Supplementary material for: Planting time shapes fall armyworm infestation dynamics and associated yield loss of maize in Bangladesh
Source: PLoS One. 2026 Apr 15;21(4):e0347125. doi: 10.1371/journal.pone.0347125 (PMC13082657; doi:10.1371/journal.pone.0347125)
Supplement: Attachment — Submitted filename: PONE-D-26-00624.docx [file pone.0347125.s007.docx]

**PONE-D-26-00624**

Planting time shapes Fall Armyworm infestation dynamics and associated yield loss of maize in Bangladesh

This study compared fall armyworm infestation and damage of maize when planted during different months in northwestern Bangladesh. The results showed that planting month had a significant effect on leaf infestation and cob damage, with planting during the winter showing lower damage than during late fall or early spring.

Several general comments: the insect name fall armyworm is usually not capitalized, as you did not capitalize the other species names. All insect species mentioned should have the scientific name plus the authority, for example, corn earworm, *Helicoverpa armigera* (Hübner). Reference names are used in line 510 rather than numbers.

Specific comments:

- For the leaf and cob infestations, I know you did “W” and diagonal transects, but did you sample the border rows or leave two or so rows unsampled at the edges? Since the plots were relatively small there could have been edge effects. Also, since FAW is invasive to Bangladesh, is the leaf feeding and cob damage similar or distinct compared to other maize pests in the region. Are there other *Spodoptera* that feed in a similar fashion or even younger *Helicoverpa*?
- For cob damage, how did you differentiate fall armyworm damage from *H. armigera* damage, or do you have evidence (pheromone traps, perhaps) that *H. armigera* populations were low?
- You mentioned that data were examined for normality and only normally distributed variables were used, but those results weren’t shown and the reader doesn’t know how many of the results were not used in the total analysis.
- It’s unclear in the text, especially for the cob damage section what the numbers are in relation to in the figures. For example, in line 285 you mention the highest infestation was in March (20.3%), but the line in Fig. 6A shows damage % over 30%. Apparently in the text you are combining the treated and untreated numbers, but why? There is a significant difference between treated and untreated damage, so you show explain this in the text. This continues in the next couple of sections where you seem to combine results across years or treatments when the figures show distinct differences.
- There is no mention as to the costs of the treatments, either the seed or foliage sprays. Would these costs be prohibitive for small-holder farmers and are they cost effective? Also, do the lower yields found for January and February plantings (Table 2) support planting at this time, even though there is lower FAW damage? Finally, is there market timing in Bangladesh that supports planting and harvest at different times?
- Supplementary Fig. 1 October, November, and March plot colors are difficult to distinguish, please use other colors or patterns.
