# Supplementary material for: Planting time shapes fall armyworm infestation dynamics and associated yield loss of maize in Bangladesh
Source: PLoS One. 2026 Apr 15;21(4):e0347125. doi: 10.1371/journal.pone.0347125 (PMC13082657; doi:10.1371/journal.pone.0347125)
Supplement: Attachment — Submitted filename: PONE_Shah_FAW Planting Date_Jan2026.docx [file pone.0347125.s008.docx]

Journal: PLOS One

Date: 01/28/2026

Title: Planting time shapes Fall Armyworm infestation dynamics and associated yield loss of maize in Bangladesh

**PLOS One publication criteria:**

1. The study presents the results of original research.
2. Results reported have not been published elsewhere.
3. Experiments, statistics, and other analyses are performed to a high technical standard and are described in sufficient detail.
4. Conclusions are presented in an appropriate fashion and are supported by the data.
5. The article is presented in an intelligible fashion and is written in standard English.
6. The research meets all applicable standards for the ethics of experimentation and research integrity.
7. The article adheres to appropriate reporting guidelines and community standards for data availability.

**Questions in bold are from the PLOS One Reviewer Guide.**

**What are the main claims of the paper and how significant are they for the discipline?**

The main claims from the paper are that FAW infestations in maize in Bangladesh exhibit a clear seasonal pattern, with peak infestations and cob damage occurring in February-March and declining infestations in December. It also states that a IPM management program of cyantraniliprole seed treatments + SfMNPV + emamectin benzoate + spinosad are effective at maintaining low infestations compared to untreated plots when infestations are high. The authors also report that seasonal infestations significantly influence grain yield and losses and highlight the necessity of planting time and management strategies to control this pest. The paper has applied and regional relevance, specifically for growers in Bangladesh who are managing invasive FAW populations.

**Are the claims properly placed in the context of the previous literature? Have the authors treated the literature fairly?**

Yes

**Do the data and analyses fully support the claims? If not, what other evidence is required?**

For the most part, see comments below.

***PLOS One* encourages authors to publish detailed protocols and algorithms as supporting information online. Do any particular methods used in the manuscript warrant such treatment? If a protocol is already provided, for example for a randomized controlled trial, are there any important deviations from it? If so, have the authors explained adequately why the deviations occurred?**

n/a

**If the paper is considered unsuitable for publication in its present form, does the study itself show sufficient potential that the authors should be encouraged to resubmit a revised version?**

Yes.

**Are original data deposited in appropriate repositories and accession/version numbers provided for genes, proteins, mutants, diseases, etc.?**

N/a

**Does the study conform to any** [**relevant guidelines**](https://journals.plos.org/plosone/s/best-practices-in-research-reporting#loc-reporting-guidelines-for-specific-research-resources-and-study-types) **such as CONSORT, MIAME, QUORUM, STROBE, and the** [**Fort Lauderdale agreement**](https://www.sanger.ac.uk/wp-content/uploads/fortlauderdalereport.pdf)**?**

N/a

**Are details of the methodology sufficient to allow the experiments to be reproduced?**

Yes

**Is any software created by the authors freely available?**

n/a

**Is the manuscript well organized and written clearly enough to be accessible to non-specialists?**

Yes

**Is it your opinion that this manuscript contains an** [**NIH-defined experiment of Dual Use concern**](https://grants.nih.gov/grants/guide/notice-files/NOT-OD-13-107.html)**?**

No

**Additional Comments/Notes**

- In text, fall armyworm does not require capitalization.
- Generic chemical names – cyantraniliprole, emamectin benzoate, and spinosad – do not require capitalization in text.
- Line 70: and stalk borer.
- Line 134: what was the larval stage at each application? Fawligen is ineffective past the 4^th^ instar.
- Lines 157 – 187: The manuscript attributes foliar and ear injury to FAW, but no information is provided on whether larvae were sampled to confirm species identity. Because other lepidopteran pests can produce similar feeding symptoms in maize, especially at the whorl and ear stages, some confirmation of FAW presence (even periodic field collections) would strengthen the species-specific conclusions. If such sampling was conducted, it should be described. If not, the authors should clarify that damage assessments were based on characteristic FAW injury and temper species-level inferences accordingly.
- Line 189-199: The study addresses an important question and the data appear potentially valuable. However, the current statistical analysis does not fully account for the split-plot design and multiple years of study. Because planting date is the main effect and treatment is the subplot effect, a mixed-model ANOVA would be more appropriate. In that case, planting date would be the main effect, treatment subplot effect, and year and replicate included as random effects. Additionally, subsamples within plots would not have to be averaged prior to analysis as the model can account for within-plot variability using replicate as a random effect.
- The results of the treatment effect are being overstated in the discussion. The primary finding is that planting date significantly affected yield, yield loss, plant, and cob numbers. In tables 1 and 2, the only time that treatment was a significant effect was in the month x treatment interaction. And in table 2, the only months with significant differences in yield were October, November, and December, with Nov and Dec having lower infestations than any other time of year. This discussion may change with the mixed-model ANOVA, but I would caution the authors to not over-emphasize the significance of treatment based on this data.
