# Supplementary material for: Planting time shapes fall armyworm infestation dynamics and associated yield loss of maize in Bangladesh
Source: PLoS One. 2026 Apr 15;21(4):e0347125. doi: 10.1371/journal.pone.0347125 (PMC13082657; doi:10.1371/journal.pone.0347125)
Supplement: Attachment — Submitted filename: Response to Reviewers.docx [file pone.0347125.s009.docx]

Date: 16 March 2026

To

Rodney N. Nagoshi, Ph.D.
Academic Editor
PLOS One

Ref: PONE-D-26-00624

Title: Planting time shapes Fall Armyworm infestation dynamics and associated yield loss of maize in Bangladesh

Dear Editor

Thank you so much for sending the comments raised by the academic editor and reviewer(s) which have allowed us to considerable improvements to the article. We are happy to inform you that we have been able to resolve almost all issues raised by the academic editor and both reviewers. If unfortunately, we have overlooked any aspects kindly let us know and we will rectify. Kindly note that all edits are available in the text in track change mode. Response for all comments raised by all reviewers and the handling editor are available below:

Journal Requirements:

When submitting your revision, we need you to address these additional requirements.

1. Please ensure that your manuscript meets PLOS ONE's style requirements, including those for file naming. The PLOS ONE style templates can be found at

<https://journals.plos.org/plosone/s/file?id=wjVg/PLOSOne_formatting_sample_main_body.pdf> and

<https://journals.plos.org/plosone/s/file?id=ba62/PLOSOne_formatting_sample_title_authors_affiliations.pdf>

Authors’ response: We meet the PLOS ONE’s style requirements including file naming.
2. In your Methods section, please provide additional information regarding the permits you obtained for the work. Please ensure you have included the full name of the authority that approved the field site access and, if no permits were required, a brief statement explaining why.

Authors’ response: Thanks for your comment and we mentioned the full name of authority in the materials and methods section.

3. Thank you for stating the following in the Acknowledgments Section of your manuscript:

“We gratefully acknowledge the Ministry of Agriculture, Government of the People’s Republic of Bangladesh, for providing financial support for this research.”

We note that you have provided funding information that is not currently declared in your Funding Statement. However, funding information should not appear in the Acknowledgments section or other areas of your manuscript. We will only publish funding information present in the Funding Statement section of the online submission form.

Please remove any funding-related text from the manuscript and let us know how you would like to update your Funding Statement. Currently, your Funding Statement reads as follows:

“The author(s) received no specific funding for this work.”

Please include your amended statements within your cover letter; we will change the online submission form on your behalf.

Authors’ response: We have removed funding statements from the acknowledgement section and amended statements included in the cover letter.

4. Thank you for stating the following financial disclosure:

“We gratefully acknowledge the Ministry of Agriculture, Government of the People’s Republic of Bangladesh, for providing financial support for this research.”

At this time, please address the following queries:

a) Please clarify the sources of funding (financial or material support) for your study. List the grants or organizations that supported your study, including funding received from your institution. 
b) State what role the funders took in the study. If the funders had no role in your study, please state: “The funders had no role in study design, data collection and analysis, decision to publish, or preparation of the manuscript.”
c) If any authors received a salary from any of your funders, please state which authors and which funders.
d) If you did not receive any funding for this study, please state: “The authors received no specific funding for this work.”

Please include your amended statements within your cover letter; we will change the online submission form on your behalf.

Authors’ response: Actually, the Ministry of Agriculture provides yearly budget to the Bangladesh Wheat and Maize Research Institute (BWMRI) like other research institutions in Bangladesh. There is no specific budget separately for this study but from the total budget of BWMRI we got the financial and material support for the study which is incorporated in the acknowledgement section.

5. When completing the data availability statement of the submission form, you indicated that you will make your data available on acceptance. We strongly recommend all authors decide on a data sharing plan before acceptance, as the process can be lengthy and hold up publication timelines. Please note that, though access restrictions are acceptable now, your entire data will need to be made freely accessible if your manuscript is accepted for publication. This policy applies to all data except where public deposition would breach compliance with the protocol approved by your research ethics board. If you are unable to adhere to our open data policy, please kindly revise your statement to explain your reasoning and we will seek the editor's input on an exemption. Please be assured that, once you have provided your new statement, the assessment of your exemption will not hold up the peer review process.

Authors’ response: We comply with the publisher’s open data policy and, during resubmission, we will revise the data availability statement to ensure that the study data are freely accessible.

6. If the reviewer comments include a recommendation to cite specific previously published works, please review and evaluate these publications to determine whether they are relevant and should be cited. There is no requirement to cite these works unless the editor has indicated otherwise. 
Authors’ response: Thank you for this guidance. We carefully reviewed the reviewer-recommended publications to assess their relevance to our study. We confirm that all citations added in the revised version were selected based on their scientific relevance and contribution to the topic.
Additional Editor Comments:

I have four primary areas of concern and also have one observation that may deserve a comment.

Major concerns:
1. This paper claims to be specific to FAW damage. However many other pests of maize are identified in Bangladesh (lines 70-73) and it is not stated in the Methods how damage specific to FAW was identified (noted by Reviewers 1 and 3). How did you distinguish plants/cobs damaged by FAW from those affected by other pests?

Authors’ response: Thank you for your valid question. In the revised version, clearly describe FAW specific damage symptoms in the methods section (leaf and cob infestation assessment subsection).

2. All three reviewers (particularly Reviewer 3) suggested modifications of the statistical analyses. Please address their concerns.

Authors’ response: The suggested modifications have been addressed and edited the revised version.

3. As noted by Reviewer 3, much is made about the impact of treatment (lines 406-418). However, from an economic perspective the most relevant metric is yield. Table 2 shows consistent yield loss between treated and untreated, but I see no analysis testing statistical significance. Please provide that comparison. The relevant question is whether any improvement in yield produced by treatment is likely to be greater than the cost of treatment. I don't expect a detailed economic analysis but it would at minimum be useful to know whether treatment significantly improves yield.

Authors’ response: Thank you for this guidance. Suggested issue has been incorporated into the revised version.

4. The conclusion (lines 534-546) recommends planting in Nov-Jan combined with treatment. Yet in Table 2, planting in October with treatment gave the highest yield. Doesn't this suggest that planting in October with treatment is the most productive strategy? Seems to me that your recommendation only makes sense for non-treatment plots where the highest yields occur in Nov-Dec. Please comment.

Authors’ response: Thank you for this direction and suggested concern has been incorporated.

Observation: I noted in Table 2 that highest yield occurred in October in treated plots but shifted to December in untreated plots. Why this shift? Do you believe it is of significance?

Authors’ response: Higher yields were obtained when IPM was combined with October planting, likely because crops planted in October experienced a longer vegetative growth period. In contrast, under the absence of FAW management, December planting produced higher yields, which corresponded with the relatively lower fall armyworm infestation observed during that period. It is noted that although the maize planting times differed, the harvesting times were nearly similar, with only a 10–20 day difference (last of May to early of June) between the earliest and the latest plantings.


[Note: HTML markup is below. Please do not edit.]

Reviewers' comments:

Reviewer's Responses to Questions

**Comments to the Author**

1. Is the manuscript technically sound, and do the data support the conclusions?

The manuscript must describe a technically sound piece of scientific research with data that supports the conclusions. Experiments must have been conducted rigorously, with appropriate controls, replication, and sample sizes. The conclusions must be drawn appropriately based on the data presented.

Reviewer #1: Yes

Reviewer #2: Yes

Reviewer #3: Partly

2. Has the statistical analysis been performed appropriately and rigorously?

Reviewer #1: Yes

Reviewer #2: Yes

Reviewer #3: No

3. Have the authors made all data underlying the findings in their manuscript fully available?

The [PLOS Data policy](http://track.editorialmanager.com/CL0/http:%2F%2Fwww.plosone.org%2Fstatic%2Fpolicies.action%23sharing/1/010f019c2127acf8-24b89760-af9d-4c4f-b7f5-93453b0fcef6-000000/meLiQlsGbkhV5IlyrqvOMlL1SYwGf0Ip2FFnyrDqAB0=248) requires authors to make all data underlying the findings described in their manuscript fully available without restriction, with rare exception (please refer to the Data Availability Statement in the manuscript PDF file). The data should be provided as part of the manuscript or its supporting information, or deposited to a public repository. For example, in addition to summary statistics, the data points behind means, medians and variance measures should be available. If there are restrictions on publicly sharing data—e.g. participant privacy or use of data from a third party—those must be specified.

Reviewer #1: Yes

Reviewer #2: Yes

Reviewer #3: Yes

4. Is the manuscript presented in an intelligible fashion and written in standard English?

PLOS ONE does not copyedit accepted manuscripts, so the language in submitted articles must be clear, correct, and unambiguous. Any typographical or grammatical errors should be corrected at revision, so please note any specific errors here.

Reviewer #1: Yes

Reviewer #2: Yes

Reviewer #3: Yes

5. Review Comments to the Author

Please use the space provided to explain your answers to the questions above. You may also include additional comments for the author, including concerns about dual publication, research ethics, or publication ethics. (Please upload your review as an attachment if it exceeds 20,000 characters)

Reviewer #1: Comments are on the attached file. Since fall armyworm is an invasive pest, the authors should describe how its feeding and damage are different from traditional maize pests in Bangladesh. The cost effectiveness of the seed treatment and foliar sprays should be mentioned and if maize harvested at the times in the study have a market.

Authors’ response: All concern comments provided in separate sheet have been responded to the revised manuscript.

Reviewer #2: General comments

This manuscript investigates how planting time influences Fall Armyworm (Spodoptera frugiperda) infestation and associated yield loss in maize in Bangladesh. The study is well structured and methodologically sound, addressing an important agronomic challenge in South Asia. The use of a three-year split-plot design with treated and untreated plots yields robust data, and the findings are presented with clear statistical analysis. Identifying a U-shaped infestation pattern and quantifying yield loss across planting months are valuable contributions to integrated pest management (IPM) strategies in the region.

Authors’ response: Thank you for your valuable comments, which we greatly appreciate and which encourage us to improve the manuscript.
However, the manuscript would benefit from being placed in a broader context within the existing literature. The impact of an otherwise strong study is slightly limited by the absence of key comparisons and some methodological justifications. Specific comments for improvement are provided below.

Abstract

Ensure the research gap is clearer. Add a sentence about why planting time studies are needed in Bangladesh.

Authors’ response: The suggested edits have been incorporated.
Lines 23-25: The description of the treatment is dense. Consider simplifying to: "... treated plots received seed treatment with Cyantraniliprole followed by foliar applications of biopesticides and insecticides; and (ii) an untreated control."

Authors’ response: The suggested edits have been incorporated.

Line 29: The term "innate leaf infestation" is confusing. Specify that it refers to "infestation in untreated control plots."

Authors’ response: The suggested edits have been incorporated.

Lines 38-39: The conclusion is vague. Strengthen it with a practical recommendation: "These findings demonstrate that adjusting planting time to November-January, combined with the described IPM strategy, is an effective approach to minimize FAW-related yield loss in Bangladesh."

Authors’ response: The suggested edits have been incorporated into the revised manuscript.

Introduction

Lines 95–102: While the rationale for studying planting time is clear, the objectives are not explicitly stated. Add a final sentence: 'The objectives of this study are to: (i) quantify the seasonal dynamics of FAW infestation across six planting months; (ii) evaluate the efficacy of an integrated pest management (IPM) strategy; and (iii) determine the associated yield losses under field conditions in northwestern Bangladesh.'

Authors’ response: The suggested edits have been incorporated into the revised manuscript.

Lines 100-102 (Gap in Literature): This section omits a highly relevant study by Cokola et al. (2024), published in PLoS ONE, which examined the effects of planting date on FAW in the Democratic Republic of Congo. Referring to this study is crucial for contextualising the global significance and originality of your findings in Bangladesh.

Authors’ response: Thank you for your suggestion. The suggested edits have been incorporated into the revised manuscript.

Materials and methods

Lines 129-143 (Treatment application protocol): The protocol is complex. A supplementary flowchart (S1 Fig.) would greatly improve clarity, showing the decision points (e.g., "IF infestation reaches 10-20%, apply SfNPV...").

Authors’ response: The suggested edits have been incorporated into the revised manuscript.


Lines 140-142: Justify the selection of these specific insecticides (Emamectin benzoate, Spinosad) briefly. E.g., "based on prior efficacy studies and their classification as moderately hazardous (WHO Class II)."

Authors’ response: The suggested edits have been incorporated into the revised manuscript.


Lines 190-199 (Statistical Analysis): The analysis is appropriate. However, specify how data normality and homogeneity of variances were checked prior to ANOVA. All statistical analyses and figures were performed using JMP statistical software, version 13 but we can see that some figures were made using R. Also, mention the specific R version used for the heatmap.

Authors’ response: The suggested edits have been incorporated into the revised manuscript.


Missing Statement: An ethics/field permission statement should be added at the end of the manuscript: "Field studies were conducted with the permission of the Bangladesh Wheat and Maize Research Institute (BWMRI) and did not involve endangered or protected species."

Authors’ response: The suggested edits have been incorporated into methods section of the revised manuscript

Results

The results are clearly presented and statistically robust, with effective visualizations (e.g. heatmaps and regression models) that demonstrate the significant differences in FAW infestation and yield outcomes between management regimes.

However, there is a significant inconsistency between the methodology description and the results presentation regarding treatment structure, which requires clarification and potentially affects the interpretation of the findings.
In the methods section (lines 129–143): The treatment is described as a sequential, integrated protocol: Seed treatment with cyantraniliprole, followed by foliar sfNPV (biopesticide), emamectin benzoate and spinosad (applied based on infestation thresholds). In the results section: Data are presented only in two aggregated categories: 'Treated' vs. 'Non-treated'.

This raises three critical questions:

1. Attribution of efficacy: Which component(s) of the multi-step protocol were most responsible for the observed suppression of FAW? Was it the seed treatment, the biopesticide, the synthetic insecticides, or a combination of these? The current presentation cannot answer this.

2. Scientific and practical value: Presenting only the combined 'Treated' result limits the study's value. A key research question, for example, is whether the biopesticide (SfNPV) could provide sufficient early-season control alone or in initial combination with the seed treatment, potentially reducing the need for synthetic insecticide sprays later on. The current design and analysis cannot address this.

3. Statistical design vs. analysis: The manuscript describes a split-plot design with 'treatment' as a subplot factor. If the 'treatment' factor had only two levels (treated/non-treated), the analysis would be correct. However, the methodology suggests that multiple distinct interventions were applied sequentially. If these were applied to the same plots, they would not be independent treatments, but rather parts of a schedule. This should be made explicit to avoid confusion.

Recommendations for revision:

In the methods: Clarify the experimental design. State explicitly: 'The treatment factor had two levels: (1) a full IPM schedule (seed treatment and sequential foliar sprays, as described) and (2) an untreated control. If any other treatment combinations were tested (e.g. seed treatment only or biopesticide only), these must be reported.

Authors’ response: Thank you for your valuable recommendation which indeed enriched the manuscript quality. The suggested edits have been incorporated into the revised manuscript.

In the discussion: Acknowledge this limitation. Add a statement such as: 'It is important to note that our study evaluated a bundled IPM package. The individual contribution of the seed treatment, biopesticide or synthetic insecticide to the overall efficacy cannot be disaggregated from our results. Future research should deconstruct this protocol to identify the most cost-effective components for smallholder farmers.'

Authors’ response: The suggested edits have been incorporated into the revised version.

For future work: Suggest that follow-up studies include factorial designs that test the components separately (e.g. seed treatment or biopesticide alone or in combination) to determine their individual and interactive effects.

Authors’ response: The suggested edits have been incorporated into the revised manuscript.
Figure and table citations are present (e.g. Fig. 1, Table 1), but the captions are either incomplete or embedded in the text.

Authors’ response: Thank you for your comment. We have been rectified as well.
Figure 5: The percentage of maize plants infested per treatment is missing. It should be two combined figures.

Authors’ response: The suggested edits have been incorporated into the revised manuscript.
On lines 246–248, when reporting the cubic model (R² = 0.456), it would be accurate to note that this indicates considerable unexplained variance. It would be accurate to briefly mention that other factors (e.g. humidity, natural enemies) also play a role.

Authors’ response: The suggested edits have been incorporated into the revised version.

Figure 7 caption: Do not combine damage intensity and % cob damage in the same figure. Instead, use a stacked bar plot with the treatments on the x-axis and the years in the legend. This will result in four combined figures.

Authors’ response: The suggested edits have been incorporated into the revised manuscript.

Discussion

Lines 322–333 (Contextualization): The discussion must reference and discuss the work of Cokola et al. (2024). Add a paragraph: “Our results align with recent findings from East Africa, where Cokola et al. (2024) reported severe FAW infestations in late-planted maize. Our study builds on this by quantifying the consequent yield loss gradient and demonstrating that an IPM regimen can mitigate, though not eliminate, the risk associated with suboptimal planting times.”

Authors’ response: Suggested edits have been incorporated into the revised version.

Missing section: Limitations. A dedicated paragraph is required to address the following:

Single-site, single-variety limitation: The findings are from one research station with one hybrid.

Authors’ response: Suggested edits have been incorporated into the revised version.

Economic feasibility: The cost-effectiveness of the recommended IPM package for smallholders was not analysed.

Authors’ response: Suggested edits have been incorporated into the revised version.

Mechanistic inference: Temperature is cited as the driver, but local FAW life-table parameters were not measured.

Authors’ response: This has been acknowledged as a limitation of the study, and future research will focus on measuring FAW life-table parameters.

Lines 413–415 (Insecticide Use): Promote Insecticide Resistance Management (IRM). Add: 'To ensure sustainability, the insecticides used here should be rotated with insecticides from different mode-of-action groups as part of a season-long IPM programme.'

Authors’ response: Suggested edits have been incorporated into the revised version.

Lines 529–546 (Conclusion): The claim that planting month is the 'single most dominant factor' (line 529) is strong, but it could be tempered to 'a dominant factor' unless it is statistically compared to all other variables.

Authors’ response: Thank you for your suggestion, edit has been incorporated as per suggested.

Reviewer #3: • In text, fall armyworm does not require capitalization.
• Generic chemical names – cyantraniliprole, emamectin benzoate, and spinosad – do not require capitalization in text.

Authors’ response: Suggested edits have been incorporated into the revised version.
• Line 70: and stalk borer.

Authors’ response: Suggested edits have been incorporated into the revised version.
• Line 134: what was the larval stage at each application? Fawligen is ineffective past the 4th instar.

Authors’ response: Suggested edits, in details, have been incorporated into the revised version.
• Lines 157 – 187: The manuscript attributes foliar and ear injury to FAW, but no information is provided on whether larvae were sampled to confirm species identity. Because other lepidopteran pests can produce similar feeding symptoms in maize, especially at the whorl and ear stages, some confirmation of FAW presence (even periodic field collections) would strengthen the species-specific conclusions. If such sampling was conducted, it should be described. If not, the authors should clarify that damage assessments were based on characteristic FAW injury and temper species-level inferences accordingly.

Authors’ response: Suggested edits, in details, have been incorporated into the revised version.
• Line 189-199: The study addresses an important question and the data appear potentially valuable. However, the current statistical analysis does not fully account for the split-plot design and multiple years of study. Because planting date is the main effect and treatment is the subplot effect, a mixed-model ANOVA would be more appropriate. In that case, planting date would be the main effect, treatment subplot effect, and year and replicate included as random effects. Additionally, subsamples within plots would not have to be averaged prior to analysis as the model can account for within-plot variability using replicate as a random effect.

Authors’ response: Thank you for your valuable comments, which have helped improve the quality of the manuscript. The suggested edits have been incorporated into the revised version.
• The results of the treatment effect are being overstated in the discussion. The primary finding is that planting date significantly affected yield, yield loss, plant, and cob numbers. In tables 1 and 2, the only time that treatment was a significant effect was in the month x treatment interaction. And in table 2, the only months with significant differences in yield were October, November, and December, with Nov and Dec having lower infestations than any other time of year. This discussion may change with the mixed-model ANOVA, but I would caution the authors to not over-emphasize the significance of treatment based on this data.

Authors’ response: Thank you for your valuable comments, which have helped improve the quality of the manuscript. The suggested edits have been incorporated into the revised version.

6. PLOS authors have the option to publish the peer review history of their article ([what does this mean?](https://track.editorialmanager.com/CL0/https:%2F%2Fjournals.plos.org%2Fplosone%2Fs%2Feditorial-and-peer-review-process%23loc-peer-review-history/1/010f019c2127acf8-24b89760-af9d-4c4f-b7f5-93453b0fcef6-000000/EpwteSdHbJdScVLD4hv8mWI2fkI6uz4Y6X3um7vTu-o=248)). If published, this will include your full peer review and any attached files.


If you choose “no”, your identity will remain anonymous but your review may still be made public.


**Do you want your identity to be public for this peer review?** For information about this choice, including consent withdrawal, please see our [Privacy Policy](https://track.editorialmanager.com/CL0/https:%2F%2Fwww.plos.org%2Fprivacy-policy/1/010f019c2127acf8-24b89760-af9d-4c4f-b7f5-93453b0fcef6-000000/ZVcCnyLFdT-eeJJEfjn0f-8dz-HnK3J5OyDQTZp2oec=248).

Reviewer #1: **Yes:** Robert L Meagher

Reviewer #2: **Yes:** Marcellin Cuma Cokola

Reviewer #3: No

[NOTE: If reviewer comments were submitted as an attachment file, they will be attached to this email and accessible via the submission site. Please log into your account, locate the manuscript record, and check for the action link "View Attachments". If this link does not appear, there are no attachment files.]
